# Draft Genome Sequence of *Massilia* sp. Strain ONC3, a Novel Bacterial Species of the *Oxalobacteraceae* Family Isolated from Garden Soil

**DOI:** 10.1128/MRA.00377-19

**Published:** 2019-08-08

**Authors:** Vincent Peta, Rachel Raths, Heike Bücking

**Affiliations:** a Biology and Microbiology Department, South Dakota State University, Brookings, South Dakota, USA; Broad Institute

## Abstract

From garden soil, we isolated and sequenced Massilia sp. strain ONC3, a new member of the *Oxalobacteraceae* within the Massilia genus. Sequence analysis showed an assembled genome size of 5,622,601 bp, with a predicted total of 5,104 protein-coding sequences, 3,194 functionally assigned genes, 2 rRNA operons, and 56 tRNAs.

## ANNOUNCEMENT

Members of the genus Massilia have been isolated from soil, air, and water samples, as well as from the plant rhizosphere and endosphere, and they are typically Gram-negative, rod-shaped aerobes (1, 2). Certain species of *Massilia* can promote plant growth through their ability to solubilize recalcitrant phosphate sources in soils (3) or through their positive impact on the colonization of plants by beneficial root symbionts such as arbuscular mycorrhizal fungi. Massilia sp. strain RK4, for example, increases root colonization by arbuscular mycorrhizal fungi and their nutritional benefits to maize plants under salt stress (4). Other *Massilia* species are highly resistant to heavy metals and have been isolated from mines (5).

We isolated a putative new species of *Massilia* from unplanted garden soil (pH 5.2) near Maxton, NC (34.6494, −79.4327). The isolate was an interesting target for whole-genome sequencing, since *Massilia* strains often show plant growth-promoting capabilities. The soil samples were dried for 3 days prior to bacterial isolation, mixed with 1× phosphate-buffered saline, streaked out several times until single colonies were isolated on R2A medium, and cultured at 30°C. Cultures were grown in R2A broth at 30°C for 2 days before genomic DNA (gDNA) was extracted using the Agencourt GenFind v2 kit (Beckman Coulter Life Sciences, Indianapolis, IN) and the protocol for bacterial gDNA extraction. The genomic library was prepared using the Illumina Nextera platform (San Diego, CA), size selected to an average fragment length of 475 bp, and sequenced using Illumina NextSeq paired-end v2 chemistry on v2.5 flow cells at 150 bp per read. The target coverage for the reads was 20×. We used the default settings of BayesHammer and SPAdes 3.13.0 (6) for quality trimming and *de novo* assembly of the 10,528,120 total reads. This resulted in 155 contigs with an *N*_50_ value of 56,531 bp (contig size range, 942 to 207,795 bp) and a total assembled size of 5,622,601 bp, with a GC content of 63.82%. Assembly quality with 40 reference proteins through BUSCO (7, 8) revealed a measured completeness (40 single-copy BUSCOs) of 100%. Genome assembly and annotation were carried out using the PATRIC 3.5.28 pipeline (9) and confirmed by Galaxy (10) and RAST 2.0 (11), which identified 5,104 protein-coding sequences, 3,194 proteins with functional assignments, 2 rRNA operons, and 56 tRNA genes.

Based on NCBI BLAST (12) searches of the 16S rRNA gene sequences, *Massilia* sp. strain ONC3 was found to have the highest similarities (98% sequence identity) with Massilia solisilvae J18 and Massilia terrae J11. The Genome-to-Genome Distance Calculator (GGDC) (13) calculates intergenomic distances using three formulas (the sum of all identities found in aligned high-scoring segment pairs divided by the total genome length and expressed as a percentage) and was used to compare the ONC3 genome to the 5 most related genomes (Massilia albidiflava DSM 17472, Massilia armeniaca ZMN-3, Massilia putida 6NM-7^T^, *Massilia* sp. strain NR 4-1, and *Massilia* sp. strain WG5 [14]). The results for the ONC3 genome relative to the other selected genomes showed a 0% match (or less than 70% of the scientific community threshold) (15, 16). This suggests that *Massilia* sp. ONC3 is a novel species within the *Massilia* genus.

### Data availability.

This genome has been deposited at DDBJ/EMBL/GenBank under the BioProject number PRJNA529408, BioSample number SAMN11265771, accession number NZ_SPUM00000000, and SRA accession number SRR9320523.

## References

[1] Vikram S, Govender N, Kabwe MH, Bezuidt O, Makhalanyane TP 2017 Draft genome sequence of *Massilia* sp. KIM isolated from South African grassland biome soils. Genom Data 13:24–26. doi:10.1016/j.gdata.2017.06.002. 28652975 PMC5476969

[2] Ofek M, Hadar Y, Minz D 2012 Ecology of root colonizing *Massilia* (*Oxalobacteraceae*). PLoS One 7:e40117. doi:10.1371/journal.pone.0040117. 22808103 PMC3394795

[3] Zheng BX, Bi QF, Hao XL, Zhou GW, Yang XR 2017 *Massilia phosphatilytica* sp. nov., a phosphate solubilizing bacteria isolated from a long-term fertilized soil. Int J Syst Evol Microbiol 67:2514–2519. doi:10.1099/ijsem.0.001916. 28853679

[4] Krishnamoorthy R, Kim K, Subramanian P, Senthilkumar M, Anandham R, Sa T 2016 Arbuscular mycorrhizal fungi and associated bacteria isolated from salt-affected soil enhances the tolerance of maize to salinity in coastal reclamation soil. Agric Ecosyst Environ 231:233–239. doi:10.1016/j.agee.2016.05.037.

[5] Feng GD, Yang SZ, Li HP, Zhu HH 2016 *Massilia putida* sp. nov., a dimethyl disulfide-producing bacterium isolated from wolfram mine tailing. Int J Syst Evol Microbiol 66:50–55. doi:10.1099/ijsem.0.000670. 26449383

[6] Nurk S, Bankevich A, Antipov D, Gurevich A, Korobeynikov A, Lapidus A, Prjibelsky A, Pyshkin A, Sirotkin A, Sirotkin Y, Stepanauskas R, McLean J, Lasken R, Clingenpeel SR, Woyke T, Tesler G, Alekseyev MA, Pevzner PA 2013 Assembling genomes and mini-metagenomes from highly chimeric reads, p 158–170. *In* Deng M, Jiang R, Sun F, Zhang X (ed), Research in computational molecular biology. Springer Berlin, Heidelberg, Germany. 10.1089/cmb.2013.0084 PMC3791033 24093227

[7] Simão FA, Waterhouse RM, Ioannidis P, Kriventseva EV, Zdobnov EM 2015 BUSCO: assessing genome assembly and annotation completeness with single-copy orthologs. Bioinformatics 31:3210–3212. doi:10.1093/bioinformatics/btv351. 26059717

[8] Mende DR, Sunagawa S, Zeller G, Bork P 2013 Accurate and universal delineation of prokaryotic species. Nat Methods 10:881–884. doi:10.1038/nmeth.2575. 23892899

[9] Wattam AR, Davis JJ, Assaf R, Boisvert S, Brettin T, Bun C, Conrad N, Dietrich EM, Disz T, Gabbard JL, Gerdes S, Henry CS, Kenyon RW, Machi D, Mao C, Nordberg EK, Olsen GJ, Murphy-Olson DE, Olson R, Overbeek R, Parrello B, Pusch GD, Shukla M, Vonstein V, Warren A, Xia F, Yoo H, Stevens RL 2017 Improvements to PATRIC, the all-bacterial bioinformatics database and analysis resource center. Nucleic Acids Res 45:D535–D542. doi:10.1093/nar/gkw1017. 27899627 PMC5210524

[10] Afgan E, Baker D, Batut B, van den Beek M, Bouvier D, Čech M, Chilton J, Clements D, Coraor N, Grüning BA, Guerler A, Hillman-Jackson J, Hiltemann S, Jalili V, Rasche H, Soranzo N, Goecks J, Taylor J, Nekrutenko A, Blankenberg D 2018 The Galaxy platform for accessible, reproducible and collaborative biomedical analyses: 2018 update. Nucleic Acids Res 46:W537–W544. doi:10.1093/nar/gky379. 29790989 PMC6030816

[11] Aziz RK, Bartels D, Best AA, DeJongh M, Disz T, Edwards RA, Formsma K, Gerdes S, Glass EM, Kubal M, Meyer F, Olsen GJ, Olson R, Osterman AL, Overbeek RA, McNeil LK, Paarmann D, Paczian T, Parrello B, Pusch GD, Reich C, Stevens R, Vassieva O, Vonstein V, Wilke A, Zagnitko O 2008 The RAST server: Rapid Annotations using Subsystems Technology. BMC Genomics 9:75. doi:10.1186/1471-2164-9-75. 18261238 PMC2265698

[12] Altschul SF, Gish W, Miller W, Myers EW, Lipman DJ 1990 Basic local alignment search tool. J Mol Biol 215:403–410. doi:10.1016/S0022-2836(05)80360-2. 2231712

[13] Meier-Kolthoff JP, Auch AF, Klenk H-P, Göker M 2013 Genome sequence-based species delimitation with confidence intervals and improved distance functions. BMC Bioinformatics 14:60. doi:10.1186/1471-2105-14-60. 23432962 PMC3665452

[14] Lou J, Gu H, Wang H, An Q, Xu J 2016 Complete genome sequence of *Massilia* sp. WG5, an efficient phenanthrene-degrading bacterium from soil. J Biotechnol 218:49–50. doi:10.1016/j.jbiotec.2015.11.026. 26656222

[15] Wayne LG, Brenner DJ, Colwell RR, Grimont PAD, Kandler O, Krichevsky MI, Moore LH, Moore WEC, Murray RGE, Stackebrandt E, Starr MP, Trüper HG 1987 Report of the ad hoc committee on reconciliation of approaches to bacterial systematics. Int J Syst Bacteriol 37:463–464. doi:10.1099/00207713-37-4-463.

[16] Tindall BJ, Rosselló-Móra R, Busse HJ, Ludwig W, Kämpfer P 2010 Notes on the characterization of prokaryote strains for taxonomic purposes. Int J Syst Evol Microbiol 60:249–266. doi:10.1099/ijs.0.016949-0. 19700448

